# Rapidly-manufactured CD276 CAR-T cells exhibit enhanced persistence and efficacy in pancreatic cancer

**DOI:** 10.1186/s12967-024-05462-7

**Published:** 2024-07-08

**Authors:** Tian Deng, Yingzhi Deng, Shih-Ting Tsao, Qinghui Xiong, Yue Yao, Cuicui Liu, Ming yuan Gu, Fei Huang, Haiying Wang

**Affiliations:** 1 Department of Research and Development, Hrain Biotechnology Co., Ltd., 1238 Zhangjiang Road, Pudong New District, Shanghai, China; 2 Regulatory Affairs Department, Hrain Biotechnology Co., Ltd., 1238 Zhangjiang Road, Pudong New District, Shanghai, China

**Keywords:** CD276, Chimeric antigen receptor, Rapidly-manufactured CAR-T, Dash CAR-T, Pancreatic cancer

## Abstract

**Background:**

Pancreatic cancer is one of the most lethal malignancies and the lack of treatment options makes it more deadly. Chimeric Antigen Receptor T-cell (CAR-T) immunotherapy has revolutionized cancer treatment and made great breakthroughs in treating hematological malignancies, however its success in treating solid cancers remains limited mainly due to the lack of tumor-specific antigens. On the other hand, the prolonged traditional manufacturing process poses challenges, taking 2 to 6 weeks and impacting patient outcomes. CD276 has recently emerged as a potential therapeutic target for anti-solid cancer therapy. Here, we investigated the efficacy of CD276 CAR-T and rapidly-manufactured CAR-T against pancreatic cancer.

**Methods:**

In the present study, CD276 CAR-T was prepared by CAR structure carrying 376.96 scFv sequence, CD8 hinge and transmembrane domain, 4-1BB and CD3ζ intracellular domains. Additionally, CD276 rapidly-manufactured CAR-T (named CD276 Dash CAR-T) was innovatively developed by shortening the duration of ex vitro culture to reduce CAR-T manufacturing time. We evaluated the anti-tumor efficacy of CD276 CAR-T and further compared the functional assessment of Dash CAR-T and conventional CAR-T in vitro and in vivo by detecting the immunophenotypes, killing ability, expansion capacity and tumor-eradicating effect of CAR-T.

**Results:**

We found that CD276 was strongly expressed in multiple solid cancer cell lines and that CD276 CAR-T could efficiently kill these solid cancer cells. Moreover, Dash CAR-T was successfully manufactured within 48–72 h and the functional validation was carried out subsequently. In vitro, CD276 Dash CAR-T possessed a less-differentiated phenotype and robust proliferative ability compared to conventional CAR-T. In vivo xenograft mouse model, CD276 Dash CAR-T showed enhanced anti-pancreatic cancer efficacy and T cell expansion. Besides, except for the high-dose group, the body weight of mice was maintained stable, and the state of mice was normal.

**Conclusions:**

In this study, we proved CD276 CAR-T exhibited powerful activity against pancreatic cancer cells in vitro and in vivo. More importantly, we demonstrated the manufacturing feasibility, acceptable safety and superior anti-tumor efficacy of CD276 Dash CAR-T generated with reduced time. The results of the above studies indicated that CD276 Dash CAR-T immunotherapy might be a novel and promising strategy for pancreatic cancer treatment.

**Supplementary Information:**

The online version contains supplementary material available at 10.1186/s12967-024-05462-7.

## Background

Pancreatic cancer is one of the most life-threatening malignancies, with the 5 year survival rate is less than 10% [1–3]. In recent years, the morbidity and mortality of pancreatic cancer have been increasing worldwide. However, surgery is still the only potential hope of cure for patients with pancreatic cancer, unfortunately, less than 20% of newly diagnosed patients are suitable for surgical resection [1, 4, 5]. Moreover, surgery is associated with high post-operative morbidity, with overall recurrence rate remains high at 70–80% [3, 6]. Therefore, highly unmet medical need in pancreatic cancer directs researchers to investigate new pancreatic cancer treatments and drugs.

CAR-T immunotherapy has emerged as a promising and effective approach for treating patients with specific hematologic malignancies [7, 8]. However, although researchers have done a great deal of work, including preclinical and clinical trials, to maximize the effectiveness of CAR-T in solid tumors, effective expansion and persistent cytotoxicity of CAR-T in vivo in solid tumors still remain a challenge due to the heterogeneity of cancer cells, tumor location, immunosuppressive tumor microenvironment [9–14]. On the other hand, all approved CAR-T cell therapy products to date involve a traditional CAR-T manufacturing process that typically takes two to 6 weeks, or even longer if CAR-T manufacturing fails [15]. Despite CAR-T cell immunotherapy has demonstrated excellent therapeutic effects, some enrolled patients still unfortunately died of disease progression prior to CAR-T infusion, mainly due to the lengthy manufacturing time of CAR-T product [16–18]. Additionally, the prolonged manufacturing time may also be accompanied by higher manufacturing costs further limit the wider applications of CAR-T therapy.

B7-H3 (also known as CD276), a member of the B7 family of immune checkpoint proteins, is a type I membrane protein with a similar sequence to the extracellular domain of PD-L1 [19, 20]. To date, the function of CD276 and the putative ligands for CD276 have not been clearly elucidated. CD276 was originally identified as a costimulatory molecule for T cell activation [21, 22]. Subsequently, several current research revealed that CD276 played a co-inhibitory role in anti-tumor immunity, and could inhibit the proliferation of T cells [20, 23]. The CD276 protein is widely expressed in human malignancies and its overexpression in tumor tissues is associated with poor prognosis, conversely, the expression of CD276 protein is limited in normal human tissues [19–21, 24–26]. In a study of 150 patients with pancreatic cancer using immunohistochemistry, 66% of patients had positive CD276 staining, and high tumor CD276 expression was independently associated with poor survival [27]. In another study of 59 patients with pancreatic cancer, CD276 was found to be highly expressed in most pancreatic cancer tissues and significantly higher than in non-cancerous tissues or normal pancreas [28]. Additionally, a study proved that CAR-T targeting CD276 could control the growth of pancreatic ductal adenocarcinoma in vitro and in orthotopic and metastatic xenograft mouse models [25]. Therefore, mounting evidence proved that CD276 has emerged as a promising therapeutic target for CAR-T therapy against solid cancers, including pancreatic cancer.

In this study, our primary focuses were on examining the safety and anti-tumor activity of CD276 CAR-T and addressing the challenge of lengthy CAR-T manufacturing time. We constructed a CAR targeting CD276 and demonstrated clear evidence of anti-tumor activity of CD276 CAR-T against many cancer cell lines in vitro. Additionally, we obtained Dash CAR-T by shortening the time of CAR-T preparation within 48–72 h. Notably, our investigations revealed that CD276-targeted Dash CAR-T maintained higher stemness and exhibited enhanced expansion ability compared with conventional CAR-T cells in vitro. Moreover, Dash CAR-T showed superior anti-tumor efficacy than conventional CAR-T in vivo pancreatic cancer model.

## Materials and methods

### Cell lines and cell culture

293T, HepG2, MHCC-97H, SK-HEP-1, PLC/PRF/5, Panc-1, MIA PaCa-2, U87MG, U251, ACHN, HT1080, MDA-MB-231, A549, HT-29 and SKOV-3 cancer lines were cultured in Dulbecco’s modified Eagle’s medium (DMEM) supplemented with 10% heat-inactivated fetal bovine serum (FBS) (Corning) and 1% penicillin/streptomycin. AsPC-1, BxPC-3, and HL-60 were cultured in RPMI 1640 (Corning) containing 10% heat-inactivated FBS. Capan-1 was cultured in IMDM (Corning) containing 20% heat-inactivated FBS. Primary human T cells were cultured in X-Vivo medium (LONZA) containing 5% Human AB Serum (GEMINI), 0.2% N-acetylcysteine (Zhejiang Cheng Yi Pharma), 1% GlutaMAX (GIBCO), 1% HEPES (GIBCO) and human 300 IU/ml IL-2 (Beijing SL Pharma). All cells except MDA-MB-231 were cultured in a humidified incubator at 37 °C and 5% CO2.

### Plasmid construction and retrovirus production

Retroviral plasmids containing CAR constructs were generated by standard molecular cloning methods. Briefly, the DNA fragment containing CD276-specific scFv derived from mouse mAb 376.96, CD8 hinge and transmembrane domain, 4-1BB and CD3ζ intracellular domains, generated by overlapping polymerase chain reaction (PCR), then subcloned into the MSGV vector. CD19 CAR control was constructed by substituting CD276 scFv sequence with CD19 scFv derived from mouse mAb FMC63. Retrovirus was produced by transient transfection of 293T cells with a three-plasmid system. Supernatant containing the retrovirus was collected 48 and 72 h after transfection, and filtered with 0.45 mm filters.

### Activation and transduction of human T cells

Peripheral blood mononuclear cells (PBMCs) obtained from healthy donors were thawed immediately before use. T cells from PBMCs were isolated and activated with CTS^™^ Dynabeads^™^ CD3/CD28 (Gibco) and then transduced with retroviral supernatants in RetroNectin-precoated (Takara) plates. The plates were then centrifuged at 2000 g for 2 h at 32 °C. After removal of the supernatant, 5 × 10^6^ activated T cells were plated, centrifuged at 1000 g for 10 min and incubated overnight. T cells were then collected and expanded in suitable containers for culture prior to being used in in vitro functional assays.

### Dash *CAR*-T manufacturing

The CAR-T cell manufacturing process including T cells activation, transduction with CAR construct, CAR-T cells extended culture and cryopreservation. Dash CAR-T and conventional (Conv.) CAR-T expressed the same validated CAR construct, but manufactures were different (Fig. 2a). Briefly, both Dash CAR-T and conventional CAR-T were generated with the strategy that T cells activation was carried out first for 24 h, then followed by cells activation concurrent with CAR construct transduction for the next 24 h. Subsequently, 48 h Dash CAR-T cells were harvested without extended culture and 72 h Dash CAR-T cells were harvested after extended culture for 24 h. Conventional CAR-T cells were obtained by extended culture of CAR-T cells for at least 4–5 days. All cells were cryopreserved before functional assays.

### Flow cytometric analysis

At harvest or upon thawing, PBMCs and final cell samples were put into flow cytometric tubes set into NT (unstaining) tubes, SA (second antibody) tubes and Sample tubes. Cells of Sample tubes were stained using the first antibodies for 30 ± 5 min, while cells of unstaining tubes mixed with 200 μl PBS were stored at 4 ℃. Then cells of SA tubes and Sample tubes were washed with PBS, incubated with second antibodies for 30 ± 5 min and resuspended in PBS. Flow Cytometric Analysis was performed on Flow Cytometer (SONY, SA3800) and a representative gating strategy was set to identify T-cell subsets. Briefly, cell population was selected and identified by plotting FSC vs. SSC. All dead cells were excluded by plotting SSC vs. Viability and living cells were selected. The CD3^+^ gate was adjusted to include CAR-T cells. Similarly, through the selected gate, CAR^+^ cells population or other T-cell immunophenotypes were further plotted and selected. CD276 CAR^+^ expression was detected by incubation with soluble Biotin-labeled human CD276-Fc fusion protein followed by staining with labeled Streptavidin.

The following first antibodies were used for cell surface staining: Pacific Blue anti-human CD3 Antibody (Biolegend); FITC anti-human CD45RO Antibody (Biolegend); PerCP/Cyanine 5.5 anti-human CD62L Antibody (Biolegend); PE/Cy7 anti-human PD-1 Antibody (Biolegend); PE anti-human TIM3 Antibody (Biolegend); APC anti-LAG-3 Antibody (Biolegend); FITC anti-human CD69 Antibody (Biolegend); Brilliant Violet 421TM anti-human CD25 Antibody (Biolegend); FMC63 (CD19)-BIO Antibody (Bioswan). The following second antibodies were used: viability Dye (Invitrogen); BV421 Streptavidin (BD); PE Streptavidin (Biolegend).

### Cytotoxicity assay

The ability of CAR-T to kill tumor target cells expressing CD276^+^ or CD276^−^ was evaluated using Luciferase-based cytotoxicity assay. Upon thawing, CAR-T cells were counted by Cell Counter (Chemometec) and cultured in X-VIVO15 complete medium supplemented with recombinant IL-2 (300 IU/ml) for 2 days in a CO2-incubator (Thermo Fisher). Tumor target cells were engineered to stably express Luciferase-GFP (LUC-GFP) and seeded into 96-well plates in 100 μl culture medium. CAR-T cells were co-cultured with LUC-GFP-expressing tumor target cells in 96-well plates for 16–72 h in the cell culture incubator. Following coculture, 50 μl Bright-Glo solution was added to the cells. Luminescence was measured after a 10–15 min incubation using the Microplate Reader. The CAR-T cells’ repetitive tumor killing potential was evaluated by the repetitive tumor challenge assays that medium with fresh LUC-GFP-expressing tumor target cells were replaced shortly (T cells were left behind).

### Cytokine release assays

T cells and target cells were co-cultured in 200 μl culture medium in 96-well round-bottom plate at a 1:1 E:T ratio (4 × 10^5^ T cells and 4 × 10^5^ target cells per well) for 24 h in a humidified incubator at 37 °C supplemented with 5% CO_2_. The co-incubated supernatants were collected for cytokine measurements of interferon (IFN)-γ concentrations using enzyme-linked immunosorbent assay (ELISA) kits (BD Biosciences) according to the manufacturer’s instructions.

### In vivo* models*

6–8 weeks old immunodeficient NOG (NOD.Cg-PrkdcscidIl2rgtm1Sug/ShiJic) mice inoculated with the human pancreatic cancer cell line Capan-1 were used to assess the anti-tumor activity of CAR-T cells in vivo. Briefly, NOG mice were injected subcutaneously with 5 × 10^6^ Capan-1 cells with 100 μl IMDM mixed with 50% matrix gel in the flank to develop the pancreatic cancer model. When the tumor volume reached about 80 mm^3^ on day 5 after tumor inoculation, 27 tumor-bearing mice were randomly assigned to 9 groups (3 in each group) according to body weight. Subsequently, different doses of CAR-T cells or vehicle were infused intravenously into mice. The tumor volume and animal weight were continuously monitored and measured twice a week, and the survival status of the mice was recorded. The tumor volume was calculated by the formula: volume = 0.5 × A × B^2^, where A and B were the long diameter and short diameter of the tumor respectively. To evaluate the CAR-T cell persistence in vivo, about 100 μl of anticoagulant peripheral blood was harvested through orbit for flow cytometry analysis every week.

### Statistical analysis

Statistical analysis was conducted using GraphPad Prism software. The graphs represent the mean value ± SEM, unless otherwise indicated. Statistics of mRNA expression of 376.96 scFv were conducted using one-way ANOVA analysis. P-values < 0.05 were considered statistically significant.

## Results

### CD276 CAR-T showed effective anti-tumor efficacy against pancreatic *cancer* cells and multiple solid tumor cell lines

**Fig. 1:**
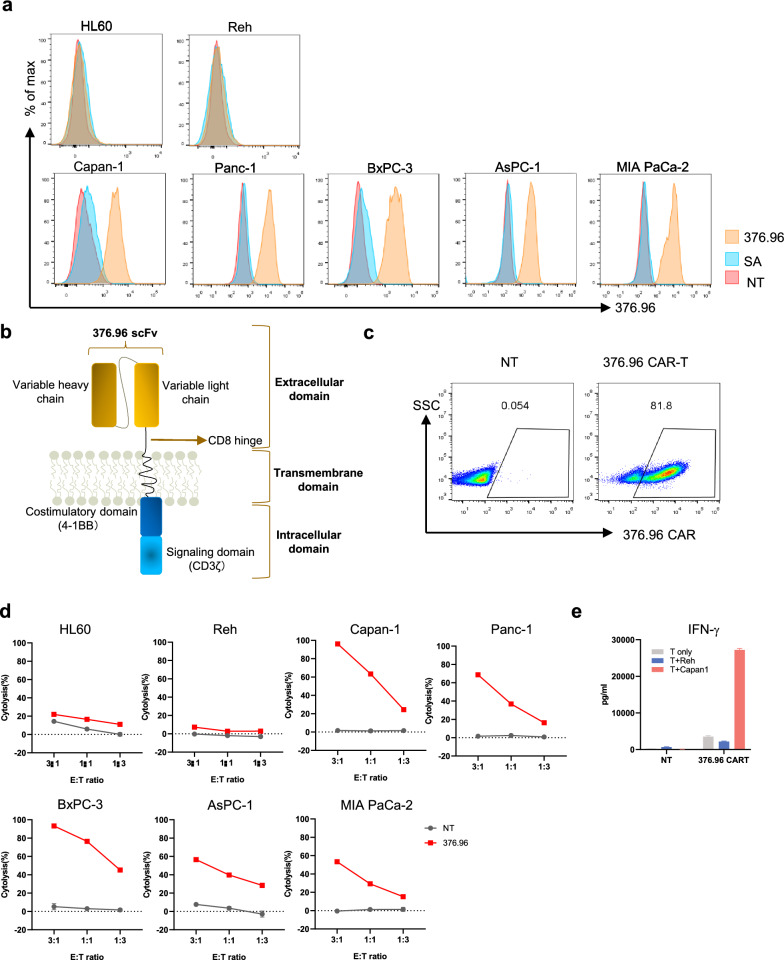
CD276 CAR-T displayed excellent anti-tumor efficacy against pancreatic cancer cells in vitro. **a**. The expression of CD276 antigen on different human tumor cell lines. 376.96 (CD276 positive antibody), SA (second antibody), NT (unstaining).**b**. Schematic of CAR design. **c**. Percentage of CAR^+^ cells of CD276 CAR-T detected at harvest and represented as flow cytometry plots. NT (no transduced) cells were as control. **d**. Killing efficiency of CD276 CAR-T cells and control NT cells toward hematologic tumor cell lines or pancreatic cancer cell lines at different E: T ratio. **e**. IFN-γ release in the supernatant of co-culture of CAR-T cells or NT cells with target cells for 24 h at a 1:1 E: T ratio. HL60, the human promyelocytic leukemia cell line; Reh, the human acute lymphoblastic leukemia cell line; Capan-1, Panc-1, BxPC-3, AsPC-1 and MIA PaCa-2, the human pancreatic cancer cell lines

Here, we first tested CD276 antigen expression by flow cytometry and found pancreatic cancer cell lines showed positive expression of CD276 while hematologic tumor cell line like Reh and HL60 exhibited negative expression of CD276 (Fig. 1a). Next, we constructed the CD276 CAR structure using the 376.96 scFv, CD8 hinge and transmembrane domain, 4-1BB and CD3ζ intracellular domains, and prepared CAR-T by infecting activated T cells with packaged retroviruses (Fig. 1b). Extended culture for 5 days after infection, we detected more than 80% CAR^+^ expression on T cells (Fig. 1c). Then, we performed functional evaluation of CD276 CAR-T during co-culture with CD276^+^ target cells or CD276^−^ target cells. As shown in Fig. 1d, CD276 CAR-T was highly effective against multiple pancreatic cancer cell lines at different E: T ratio while the killing efficiency was low in eradicating hematologic tumor cell lines, indicating a CD276-specific cytotoxicity. Moreover, after exposure to Capan-1 cells for 24 h significantly enhanced the production of IFN-γ in CD276 CAR-T relative to Reh cells (Fig. 1e). As a pan-cancer antigen, we found that CD276 was highly expressed in many cancer cell lines (Supplementary Fig. 1a). At different E:T ratios, CD276 CAR-T showed high killing efficiency (cytolysis > 80%) against different human cancer cell lines, including liver cancer, glioma, renal cancer, fibrosarcoma, breast cancer, ovarian cancer and colon cancer (Supplementary Fig. 1b).

### CD276 dash *CAR*-T manufactured with reduced time maintained higher stemness compared with conventional *CAR*-T

**Fig. 2:**
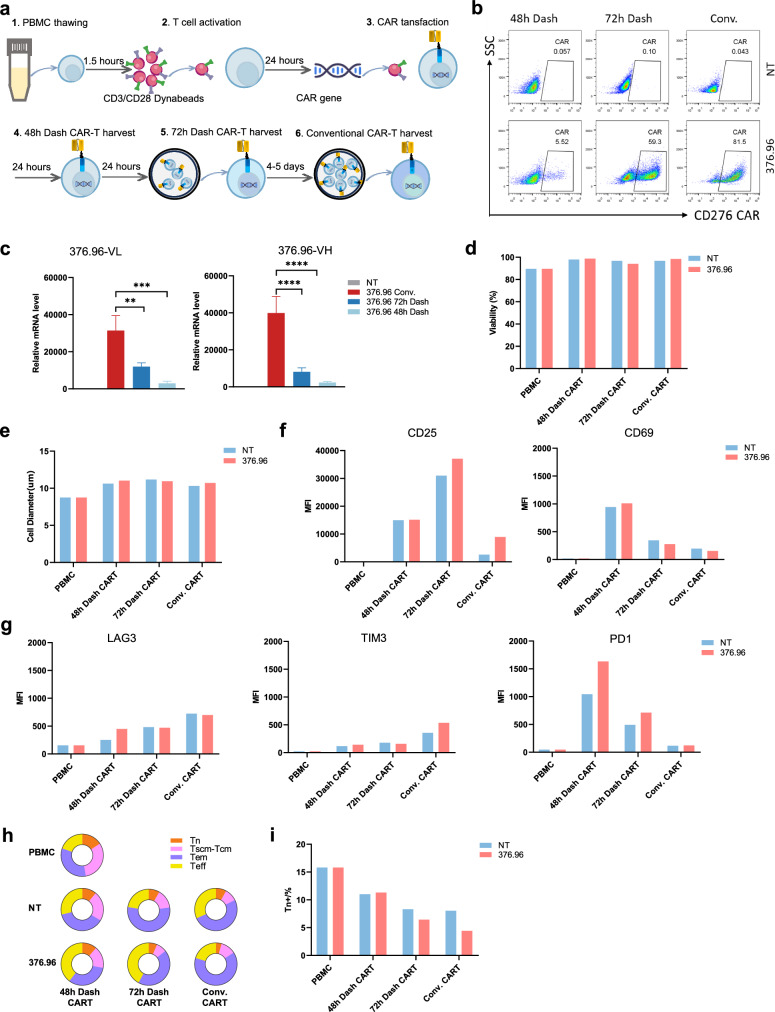
The immunophenotypes of CD276 Dash CAR-T manufactured with reduced time in vitro. **a**. Process flow diagram for manufacturing of Dash CAR-T cells and Conv. CAR-T cells. Dash CAR-T cells include 48h Dash CAR-T cells and 72h Dash CAR-T cells. **b**. Percentage of CAR^+^ cells of CD276 Dash CAR-T cells and Conv. CAR-T cells detected at harvest and represented as flow cytometry plots. **c**. The mRNA expression of the single chain variable fragment (scFv) from the anti-CD276 376.96 monoclonal antibody. Left is the single variable VL domain, Right is the single variable VH domain. **d**. Cell viability was measured during the manufacture of Dash CAR T cells and Conv. CAR T cells. **e**. Cell diameter was measured during the manufacture of Dash CAR T cells and Conv. CAR T cells. **f**. MFI of T cell activation markers CD25 and CD69 expression on the PBMC, 48h Dash CAR-T, 72h Dash CAR-T or Conv. CAR-T cells surface at harvest. **g**. MFI of T cell exhaustion markers LAG3, TIM3 and PD1 expression on the PBMC, 48h Dash CAR-T, 72h Dash CAR-T or Conv. CAR-T cells surface at harvest. **h**. CD45RO/CD62L expression in CAR-T cells indicating the proportion of CAR-T cell subtypes including Tn (CD45RO^−^/CD62L^+^), Tscm-Tcm (CD45RO^+^/CD62L^+^), Tem (CD45RO^+^/CD62L^−^) and Teff (CD45RO^−^/CD62L^−^) in PBMC and at CAR-T harvest. **i**. Proportion of Tn cells in PBMC, 48h Dash CAR-T, 72h Dash CAR-T or Conv. CAR-T cells at harvest

To reduce CAR-T manufacturing time, we use the DASH platform to harvest CD276 48 h and 72 h Dash CAR-T within 48–72 h by concurrently activating and transducing T cells for 48 h (Fig. 2a). We compared the difference in cell parameters and immunophenotypes between CD276 Dash CAR-T and conventional CAR-T (Conv. CAR-T). As shown in Fig. 2b, c, the expression of 376.96 CAR^+^ protein and 376.96 scFv mRNA in conventional CAR-T was higher than that of CD276 Dash CAR-T. Both CD276 Dash CAR-T and conventional CAR-T demonstrated great cellular health indicted by cell viability (Fig. 2d). In addition, in conventional CAR-T, T cell activation markers, including the expression of CD25 and CD69 in T cells and cell diameter, were reduced (Fig. 2e, f). Moreover, cell exhaustion markers were detected and the PD1 expression was significantly reduced in CD276 conventional CAR-T compared to Dash CAR-T (Fig. 2g). It could be plausible as the CD276 Dash CAR-T was harvested after CAR transduction and T cell activation without prolonged culture in vitro. Notably, we found that CD276 Dash CAR-T, particularly the CD276 48h Dash CAR-T, exhibited a less-differentiated phenotype similar to the starting material PBMC at harvest and maintained a higher proportion of Tn cells compared to conventional CAR-T, indicating higher stemness of CD276 Dash CAR-T (Fig. 2, i). Besides, we observed similar changes in the cell parameters and immunophenotypes of CD276 Dash CAR-T from another healthy donor’ PBMC (Supplementary Fig. 2). Together, these results showed that it was feasible to reduce the time to manufacture CD276 Dash CAR-T.

### The cytotoxicity and expansion ability of CD276 dash CAR-T were different from conventional CAR-T in vitro

**Fig. 3:**
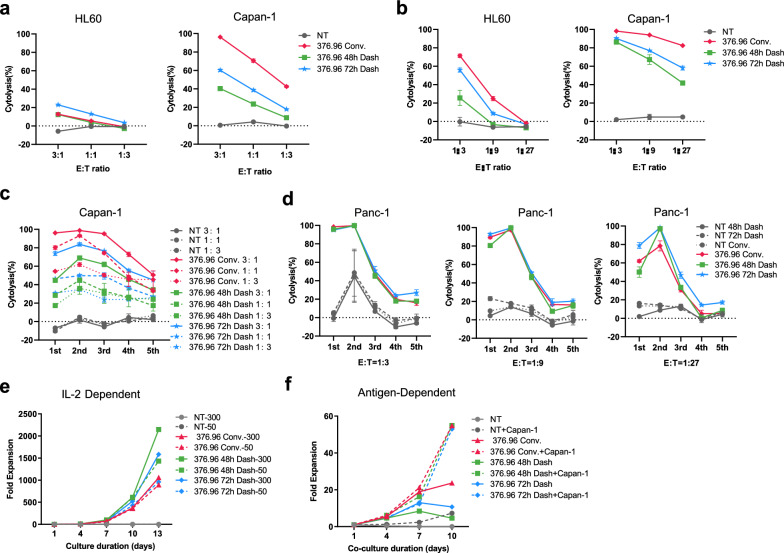
The target-eliminating efficacy and expansion ability of CD276 Dash CAR-T in vitro. **a**. Cytotoxicity of Dash CAR-T cells and Conv. CAR-T cells on CD276^−^ HL60 target cells or CD276^+^ Capan-1 target cells after co-culture for 16 h at different E: T ratio. **b**. Cytotoxicity of Dash CAR-T cells and Conv. CAR-T cells on CD276^−^ HL60 target cells or CD276^+^ Capan-1 target cells after co-culture for 72 h at different E: T ratio. **c.** Cytotoxicity of Dash CAR-T cells and Conv. CAR-T cells against CD276^+^ Capan-1 cells in rechallenges of target cells at different E: T ratio. **d**. Cytotoxicity of Dash CAR-T cells and Conv. CAR-T cells against CD276^+^ Panc-1 cells in rechallenges of target cells at different E: T ratio. **e**. CAR-T cell expansion during co-culture with different doses of IL-2. **f**. CAR-T cell expansion during co-culture with CD276^+^ Capan-1 target cells

We proceeded to compare the functional assessment of CD276 Dash CAR-T and conventional CAR-T in vitro. Compared with CD276 Dash CAR-T, conventional CAR-T induced more cytotoxic effects against CD276^+^ Capan-1 target cells after co-culture with Capan-1 cells at different E: T ratios for 16 h (Fig. 3a) and 72 h (Fig. 3b), and after rechallenge of Capan-1 cells with different E: T ratios (Fig. 3c). On the contrary, we further evaluated the killing efficacy against CD276^+^ Panc-1 cells and found that CD276 Dash CAR-T showed greater cytotoxicity in rechallenge of Panc-1 target cells at a lower 1:27 E: T ratio (Fig. 3d and Supplementary Fig. 4a). CD276 Dash CAR-T also showed greater cytotoxicity in rechallenge of ovarian cancer cell SKOV3 at a lower 1:9 and 1:27 E: T ratios (Supplementary Fig. 3 and Supplementary Fig. 4b). In addition, during co-culture with different doses of IL-2, we observed that CD276 Dash CAR-T had a higher IL-2-dependent expansion capacity than conventional CAR-T (Fig. 3e). Dash CAR-T cells underwent an approximately 2000-fold expansion during a 13 day culture, while the corresponding conventional CAR-T had only a 1057-fold amplification. The expansion ability of CAR-T, no matter CD276 Dash CAR-T or conventional CAR-T, was obvious high during co-culture with Capan-1 target cells (Fig. 3f). However, CD276 Dash CAR-T demonstrated antigen-dependent expansion ability comparable to conventional CAR-T. Whatever, CD276 Dash CAR-T exhibited greater cytotoxicity at a low E:T ratio and enhanced IL-dependent expansion ability compared with conventional CAR-T in vitro.

### CD276 dash CAR-T demonstrated enhanced pancreatic tumor control and improved expansion ability compared with conventional CAR-T in vivo.

**Fig. 4:**
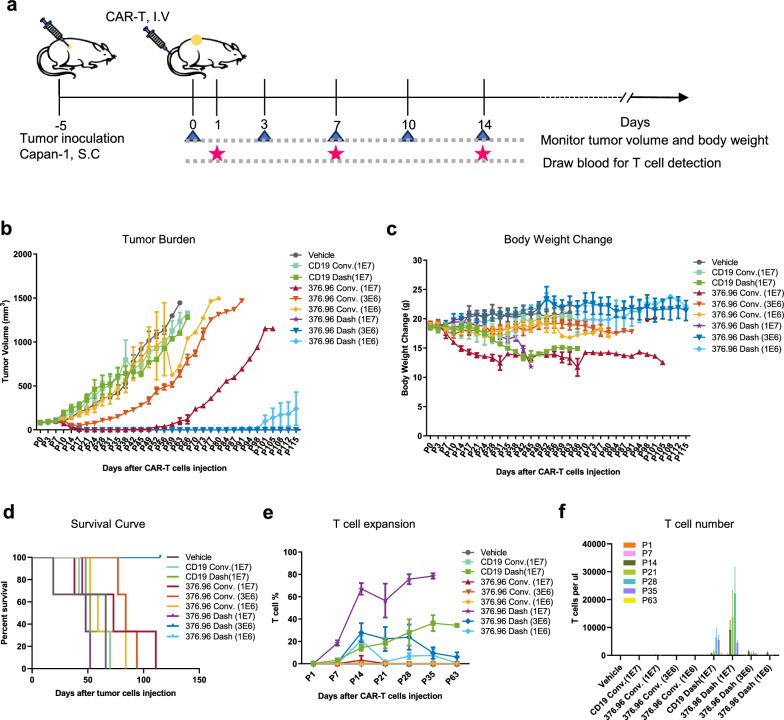
CD276 Dash CAR-T showed superior anti-tumor efficacy in vivo pancreatic cancer model. **a**. The flowchart of treatment including subcutaneous injection of Capan-1 tumor cells and vain-tail injection of CAR-T cells. **b**. Capan-1 cell engraftment NOG mice were given high dose (1 × 10^7^/mice, n = 3), medium dose (3 × 10^6^/mice, n = 3) and low dose (1 × 10^6^/mice, n = 3) 376.96 Dash CAR-T or Conv. CAR-T along with CD19 Dash CAR-T and CD19 Conv. CAR-T and vehicle groups as control. Tumor burden was monitored by tumor volume once every 3–4 days. **c**. Body weight change of the mice in each group. **d**. Overall survival of the mice in each group. **e**. CAR-T cell percentage in peripheral blood of mice was analyzed by flow cytometry. **f**. CAR-T cell number in peripheral blood of mice was analyzed by flow cytometry.

Animal experiments are crucial for the development of new human therapeutic agents because it can provide important and precise information about efficacy and safety of CAR-T cells. To further evaluate the anti-tumor efficacy of CD276 Dash CAR-T on pancreatic cancer in physiological environment, we constructed a NOG mouse xenograft model with subcutaneous injection of Capan-1 cells. The treatments were given on Day 5 after tumor inoculation (average tumor size = 80 mm^3^), and then the tumor volume, body weight and peripheral blood T cells of mice were monitored and analyzed (Fig. 4a). The anti-tumor effect of CD276 Dash CAR-T at the high doses (1E7 CAR-T cells) was similar to that of conventional CAR-T, but the mice died at about 40 days after CAR-T injection due to the rapid and massive expansion of CD276 Dash CAR-T in vivo (Fig. 4b–d). Encouragingly, CD276 Dash CAR-T showed superior tumor-control ability than conventional CAR-T when administered at medium doses (3E6 CAR-T cells) and low doses (1E6 CAR-T cells). In detail, both the medium- and low-dose CD276 Dash CAR-T groups achieved complete tumor eradication at 21 days after administration and remained tumor-free. The medium-dose CD276 Dash CAR-T group had no tumor recurrence until the end of the trial (our experiment end on the day 115 after the treatment), and the low-dose Dash CAR-T group had slow tumor growth in some mice on day 91 after administration. In contrast, the medium-dose conventional CAR-T group suppressed the tumor for a short period, after which the tumor continued to grow, while the low-dose conventional CAR-T group showed continuous tumor growth from the beginning of treatment. Besides, the control groups, including CD19 Dash CAR-T, CD19 conventional CAR-T and Vehicle, failed to suppress the tumor growth. In addition, the mice in the medium-dose and low-dose groups exhibited a normal healthy state and their body weights remained stable (Fig. 4c). By analyzing peripheral blood T cells in mice, we found that the peak of peripheral blood T cell expansion occurred at 14 days after administration, and CAR-T cells in the blood of mice injected with Dash CAR-T significantly expanded and persisted compared with conventional CAR-T (Fig. 4e, f). The results of in vivo anti-tumor evaluation concurrent with CAR-T expansion detection showed that CD276 Dash CAR-T demonstrated enhanced anti-tumor efficacy, improved expansion and persistence in vivo functional validation.

## Discussion

In the present study, we developed CD276 Dash CAR-T manufactured with less time and presented evidence that Dash CAR-T exhibited higher stemness and enhanced pancreatic tumor control compared to conventional CAR-T in vitro and in vivo. The result is important because it indicates that rapidly-manufactured CAR-T targeting CD276 for pancreatic cancer is feasible and promising by providing precise functional validation in preclinical studies.

Despite its successes in hematological malignancies, CAR-T therapy encounters challenges in addressing solid cancers. The key point of CAR-T therapy for solid tumor is the selection of safe and effective targets. Recent mounting evidence have proved that CD276 is expressed in a variety of solid cancer cells and is an excellent pan-cancer target [25, 29–31]. Here we found that CD276 CAR-T could efficiently kill multiple tumor cells in vitro and eradicate pancreatic cancer cells in vivo.

Additionally, CAR-T therapeutic effect not only depends on the CAR structure, including antigen recognition region, but also relies on the in vitro manufacturing process. Extended culture after T cell activation and CAR transduction is the essential manufacturing process for conventional CAR-T manufacture, and culture period for CAR-T expansion is generally 7–14 days in most protocols [32]. Here we introduced Dash CAR-T with extended culture for no more than 24 h, and compared the cellular immunophenotypes of Dash CAR-T and conventional CAR-T. Both CD276 Dash CAR-T and conventional CAR-T demonstrated comparable great cellular health, while higher levels of cell activation and exhaustion were observed in Dash CAR-T, which likely due to the early harvest of Dash CAR-T after T cell activation and γ-retrovirus transduction during manufacturing process [33–35]. Besides, although 48 h Dash CAR-T without extended culture had lower CAR^+^ expression, this result was understandable given the high proportion of CAR^+^ cells observed in 72 h Dash CAR-T and conventional CAR-T with extended culture, suggesting a gradual recovery of CAR^+^ expression. Overall, these results demonstrated that generating Dash CAR-T by shortening the duration of ex vitro culture was feasible.

Research has found that T cells undergo progressive differentiation during ex vitro culture, hence longer CAR-T manufacturing time may accelerate cell differentiation, resulting in less naive-like and central memory cells [36, 37]. These published results were reaffirmed in our findings. CD276-targeting Dash CAR-T manufactured within 48–72 h maintained a less-differentiated phenotype compared to conventional CAR-T prepared over 7–8 days. Less-differentiated T cells are associated with enhanced anti-tumor efficacy and improved clinical outcome [37–40]. Therefore, rapid CAR-T manufacture has recently attracted considerable attention and emerging evidence have shown that rapidly-manufactured CAR-T was involved in less differentiated state, improved expansion ability and superior anti-tumor efficacy in vivo [18, 36, 37, 41]. However, these studies all focused on hematological malignancies, and the efficacy of these rapidly-manufactured CAR-T in solid cancer remains to be determined. Here, in a solid tumor-bearing mouse model, we found that both CD276-targeted conventional CAR-T and Dash CAR-T exhibited remarkable pancreatic cancer-control ability. Notably, Dash CAR-T with a higher proportion of T naïve cells prior to infusion showed superior anti-tumor efficacy concurrent with higher and persistent CAR-T cells expansion in vivo.

Besides, some studies have found that CAR-T cells harvested early exhibited enhanced potency and persistence against hematological malignancies at low E: T ratios [18, 37, 41]. Our results confirmed this. On the one hand, we found that at a lower E: T ratio of 1:27 in vitro, Dash CAR-T demonstrated an obviously enhanced cytotoxicity to pancreatic cancer cells upon repetitive stimulation compared to conventional CAR-T. In contrast, no greater anti-tumor benefit with Dash CAR-T observed at high E: T ratios compared to conventional CAR-T. On the other hand, when tested in vivo, Dash CAR-T showed exceptional anti-tumor efficacy at a lower dose, as evidenced by Dash CAR-T requiring only one-tenth the dose of conventional CAR-T to achieve better tumor control than conventional CAR-T.

There are still some issues that need further research. Despite promising efficacy, CAR-T cell therapy is associated with significant side effects. Although in our research, except for the high-dose group, all mice were in a normal state after injection of Dash CAR-T, but because Dash CAR-T showed stronger proliferation ability than conventional CAR-T in vivo, the clinical and toxic side effects in humans remain uncertain. Therefore, the safety and efficacy of CD276 Dash CAR-T in pancreatic cancer patients need to be further explored in the future. Nevertheless, we validated the anti-tumor effects of CD276 CAR-T and Dash CAR-T against pancreatic cancer in preclinical studies and demonstrated that CD276 Dash CAR-T may be a potential treatment for pancreatic cancer patients.

## Conclusions

In summary, we reported on a CAR-T cell directed at CD276 that showed strong activity against pancreatic cancer cells in vitro and in vivo. Furthermore, we developed a time-efficient manufacturing strategy to generate Dash CAR-T enriched for the T naïve subset, as well as augmented in vivo function in a human pancreatic tumor xenograft model, compared to conventional CAR-T. These findings suggested that CD276-targeted Dash CAR-T might be a valuable option for pancreatic cancer patients, worthy of further investigation, and provided a time-saving and high-efficacy CAR-T manufacturing approach applicable to other target CAR-T cells.

### Supplementary Information

Supplementary Material 1. Figure 1. CD276 CAR-T showed strong cytotoxic activity in multiple cancer cell lines. a. Different human tumor cell lines showed positive expression of CD276 antigen. 376.96 (CD276 positive antibody), SA (second antibody), NT (unstaining). b. Killing efficiency of CD276 CAR-T cells and control NT cells toward different human cancer cell lines at different E: T ratio. HepG2, MHCC-97H, SK-HEP-1, and PLC/PRF/5, the human hepatocellular carcinoma cell lines; U87 and U251, the human glioblastoma cell lines; MDA-MB-231, the human breast cancer cell lines; SKOV3, the human ovary carcinoma cell line; A549, the human lung cancer cell line; HT29, the human colorectal cancer cell line; ACHN, the human renal cell carcinoma cell line; HT1080, the human fibrosarcoma cell line. Figure 2. The immunophenotypes of CD276 Dash CAR-T from another healthy donor’ PBMC. a. Percentage of CAR^+^ cells within 48h Dash CAR-T, 72h Dash CAR-T and Conv. CAR-T cells at harvest. b. Cell viability was measured during the manufacture of Dash CAR-T cells and Conv. CAR-T cells. c. CD45RO/CD62L expression in CAR-T cells indicating the proportion of CAR-T cell subtypes including Tn (CD45RO^-^/CD62L^+^), Tscm-Tcm (CD45RO^+^/CD62L^+^), Tem (CD45RO^+^/CD62L^-^) and Teff (CD45RO^-^/CD62L^-^) in PBMC and at CAR-T harvest. d. Proportion of Tn cells in PBMC, 48h Dash CAR-T, 72h Dash CAR-T or Conv. CAR-T cells at harvest. e. Cell diameter was measured during the manufacture of Dash CAR-T cells and Conv. CAR-T cells. f. MFI of T cell activation markers CD25 and CD69 expression on the PBMC, 48h Dash CAR-T, 72h Dash CAR-T or Conv. CAR-T cells surface at harvest. g. MFI of T cell exhaustion markers LAG3, TIM3 and PD1 expression on the PBMC, 48h Dash CAR-T, 72h Dash CAR-T or Conv. CAR-T cells surface at harvest. Figure 3. The killing efficacy against ovarian cancer cells of CD276 Dash CAR-T. **a.** Cytotoxicity of Dash CAR-T cells and Conv. CAR-T cells against CD276^+^ SKOV3 cells in rechallenges of target cells at different E: T ratios. Figure 4. The killing efficacy of CD276 Dash CAR-T from another healthy donor’ PBMC. a. Cytotoxicity of Dash CAR-T cells and Conv. CAR-T cells against CD276^+^ Panc-1 cells in rechallenges of target cells at different E: T ratios. b. Cytotoxicity of Dash CAR-T cells and Conv. CAR-T cells against CD276^+^ SKOV3 cells in rechallenges of target cells at different E: T ratios.

## Data Availability

The datasets generated and analyzed during the current study are not publicly available due to the confidentiality agreement of Hrain Biotechnology Co., Ltd. Still, they are available from the corresponding author on reasonable request.

## Abbreviations

CAR: Chimeric antigen receptor
IL-2: Interleukin 2
PBMC: Peripheral blood mononuclear cells
NT cell: No transduced cell
SA tube: Second antibody tube
NT tube: Unstaining tube
IFN-γ: Interferon gamma
Tn: Naïve T cell
Tscm: Stem cell-like memory T cell
Tcm: Central memory T cell
Tem: Effector memory T cell
Teff: Effector T cell
Conv. CAR-T: Conventional CAR-T
Dash CAR-T: Rapidly-manufactured CAR-T
