# Supplementary figures and images for: Rapidly-manufactured CD276 CAR-T cells exhibit enhanced persistence and efficacy in pancreatic cancer
Source: J Transl Med. 2024 Jul 8;22:633. doi: 10.1186/s12967-024-05462-7 (PMC11229349; doi:10.1186/s12967-024-05462-7)

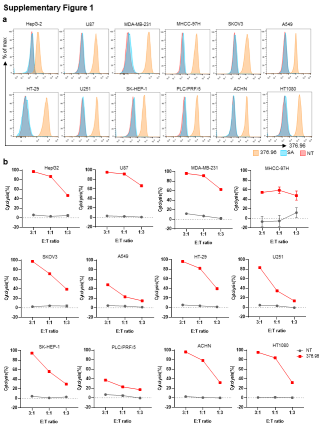

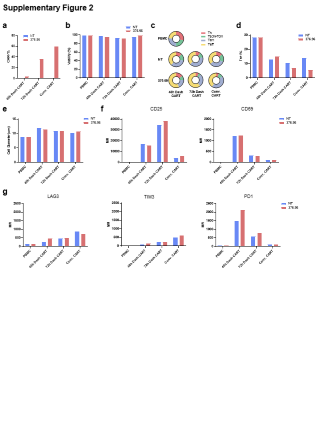

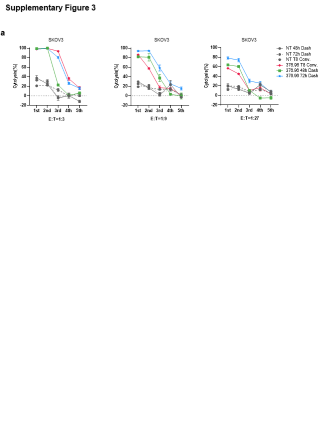

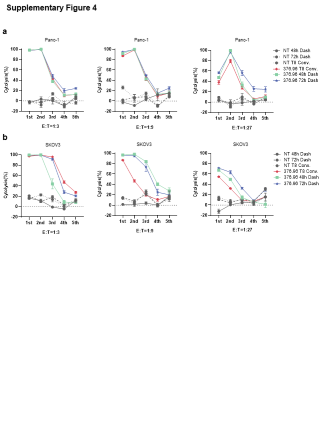

Supplement: Supplementary file 1 — Supplementary Material 1. Figure 1. CD276 CAR-T showed strong cytotoxic activity in multiple cancer cell lines. a. Different human tumor cell lines showed positive expression of CD276 antigen. 376.96 (CD276 positive antibody), SA (second antibody), NT (unstaining). b. Killing efficiency of CD276 CAR-T cells and control NT cells toward different human cancer cell lines at different E: T ratio. HepG2, MHCC-97H, SK-HEP-1, and PLC/PRF/5, the human hepatocellular carcinoma cell lines; U87 and U251, the human glioblastoma cell lines; MDA-MB-231, the human breast cancer cell lines; SKOV3, the human ovary carcinoma cell line; A549, the human lung cancer cell line; HT29, the human colorectal cancer cell line; ACHN, the human renal cell carcinoma cell line; HT1080, the human fibrosarcoma cell line. Figure 2. The immunophenotypes of CD276 Dash CAR-T from another healthy donor’ PBMC. a. Percentage of CAR+ cells within 48h Dash CAR-T, 72h Dash CAR-T and Conv. CAR-T cells at harvest. b. Cell viability was measured during the manufacture of Dash CAR-T cells and Conv. CAR-T cells. c. CD45RO/CD62L expression in CAR-T cells indicating the proportion of CAR-T cell subtypes including Tn (CD45RO-/CD62L+), Tscm-Tcm (CD45RO+/CD62L+), Tem (CD45RO+/CD62L-) and Teff (CD45RO-/CD62L-) in PBMC and at CAR-T harvest. d. Proportion of Tn cells in PBMC, 48h Dash CAR-T, 72h Dash CAR-T or Conv. CAR-T cells at harvest. e. Cell diameter was measured during the manufacture of Dash CAR-T cells and Conv. CAR-T cells. f. MFI of T cell activation markers CD25 and CD69 expression on the PBMC, 48h Dash CAR-T, 72h Dash CAR-T or Conv. CAR-T cells surface at harvest. g. MFI of T cell exhaustion markers LAG3, TIM3 and PD1 expression on the PBMC, 48h Dash CAR-T, 72h Dash CAR-T or Conv. CAR-T cells surface at harvest. Figure 3. The killing efficacy against ovarian cancer cells of CD276 Dash CAR-T. a. Cytotoxicity of Dash CAR-T cells and Conv. CAR-T cells against CD276+ SKOV3 cells in rechallenges of target cells at [file 12967_2024_5462_MOESM1_ESM.docx]
